# Supplementary material for: Cochlear implant positioning and fixation using 3D-printed patient specific surgical guides; a cadaveric study
Source: PLoS One. 2022 Jul 25;17(7):e0270517. doi: 10.1371/journal.pone.0270517 (PMC9312396; doi:10.1371/journal.pone.0270517)
Supplement: S1 Table — All data. (PDF) [file pone.0270517.s001.pdf]

| Cadaver | Medial-Lateral | Anterior-Posterior | Caudal-Cranial | Y rotation | X rotation | Z rotation |
|---|---|---|---|---|---|---|
| | X translation (mm) | Y translation (mm) | Z translation (mm) | Roll (°) | Pitch (°) | Yaw (°) |
| **Cadaver 01: Post implant right** | -0.67 | 2.68 | -3.43 | 7.91 | -1.53 | 5.74 |
| **Cadaver 01: Post implant left** | -2.72 | 0.25 | -20.3 | 19.65 | -12.43 | 6.8 |
| **Cadaver 02: Post implant right** | 1.13 | 14.33 | -8.15 | 4.87 | 3.63 | 3.87 |
| **Cadaver 02: Post implant left** | 1.26 | 4.26 | -0.73 | 4.86 | -4.24 | 0.67 |
| **Cadaver 03: Post implant right** | 1.71 | 4.57 | 1.58 | 1.5 | -7.16 | 10.42 |
| **Cadaver 03: Post implant left** | 1.68 | 2.23 | -0.32 | -0.83 | 8.97 | 5.27 |
| **Cadaver 04: Post implant right** | 1.06 | 1.68 | -4.3 | 11.41 | -7.94 | -0.56 |
| **Cadaver 04: Post implant left** | 1.1 | 1.57 | 3.38 | 3.22 | -12.75 | -2.27 |
| **Cadaver 05: Post implant right** | 1.87 | 0.81 | -4.18 | 5.04 | -2.49 | 0.22 |
| **Cadaver 05: Post implant left** | 1.81 | 1.25 | -5.76 | 5.94 | -5.09 | -1.28 |
| **Cadaver 06: Post implant right** | 0.88 | -8.13 | -4.29 | 4.29 | -2.2 | -3.12 |
| **Cadaver 06: Post implant left** | 0.84 | -2.44 | -3.25 | 3.49 | -3.36 | -3.55 |
| **Cadaver 07: Post implant right** | 1.97 | 3.99 | -1.88 | -0.1 | -12.55 | 3.9 |
| **Cadaver 07: Post implant left** | 2.45 | 1.87 | -0.36 | 4.11 | -5.75 | 2.03 |

| | | | | | | |
|---|---|---|---|---|---|---|
| **Cadaver 08: Post implant right** | -1.49 | 9.24 | -10.67 | 17.45 | -23.73 | 11.07 |
| **Cadaver 08: Post implant left** | -1.24 | 7.16 | -10.96 | 12.33 | -21.5 | 4.12 |
| **Cadaver 09: Post implant right** | 2.25 | 1.32 | 2.93 | 2.79 | -4.43 | 1.92 |
| **Cadaver 09: Post implant left** | 3.48 | 1.4 | 2.31 | 1.73 | -5.24 | 7.66 |
